# Synchrotron-based multi-scale biological imaging inside biocontainment environments

**DOI:** 10.1107/S1600577526007083

**Published:** 2026-08-26

**Authors:** Mateus Borba Cardoso, Renata Santos Rabelo, Carla Cristina Polo, Lucas Sanfelici, Renan Ramalho Geraldes, Gabriel Barros Zanoni Lopes Moreno, Henrique Pavani Pereira Ramos, Gean Rossi dos Santos, Artur Clarindo Pinto, Sérgio Augusto Lordano Luiz, Harry Westfahl

**Affiliations:** a Brazilian Synchrotron Light Laboratory (LNLS), Brazilian Center for Research in Energy and Materials (CNPEM), CEP 13083-100Campinas, São Paulo, Brazil; ESRF – The European Synchrotron, France

**Keywords:** synchrotron beamlines, biocontainment, BSL-3, BSL-4, X-ray imaging, X-ray tomography, BSL-3/BSL-4

## Abstract

The Orion project is designed to be a state-of-the-art research complex that studies dangerous pathogens, located at the Brazilian Center for Research in Energy and Materials (CNPEM), in Campinas, Brazil. Orion is set to host the first Latin America Biosafety Level 4 (BSL-4) laboratory, designed for maximum biological containment. This facility will be integrated with the Brazilian synchrotron light source, SIRIUS, and will also feature Biosafety Level 2 (BSL-2) and 3 (BSL-3) laboratories equipped for biological research, as well as an advanced electron microscopy facility. Orion’s infrastructure is poised to significantly advance the understanding of pathogens and the diseases they cause while also contributing to public health surveillance and policy development. At the core of this effort are three synchrotron-based beamlines that will connect SIRIUS to Orion, enabling cutting-edge multi-scale biological imaging. These beamlines are strategically designed to provide different levels of field-of-view, penetration power and resolutions: (1) SIBIPIRUNA, dedicated to cryo-nanotomography using soft X-ray absorption contrast for cellular imaging; (2) TIMBÓ, mainly focused on cryo-nanotomography through coherent X-ray phase-contrast for tissue studies; and (3) HIBISCO, devoted to small living animal imaging using hard X-ray phase-contrast microtomography. This unique interplay between synchrotron-based imaging techniques within biocontainment areas will undoubtedly enhance pathogen research and bioimaging, driving scientific advancements with far-reaching implications for global health.

## Abstract 2

Orion, the first BSL-4 synchrotron-based research complex, advances pathogen research by integrating cutting-edge, multi-scale imaging to enhance the understanding of infectious diseases and inform global health policies.

## 1. Introduction

Studying haza­rdous pathogens such as SARS-CoV-2, monkeypox virus (mPOX) and Ebola has become increasingly critical in the face of emerging global health threats. Owing to their high transmissibility and potential to cause severe disease, these pathogens require secure environments for manipulation, particularly Biosafety Level 3 (BSL-3) and Biosafety Level 4 (BSL-4) laboratories. Such structures protect researchers and the surrounding environment from accidental exposure while enabling studies that support the development of vaccines, therapeutics and diagnostic tools (Richardson *et al.*, 2020 ▸).

In parallel, the ability to visualize pathogens and infected biological systems across multiple length scales, from molecular structures to host-cell and tissue interactions, is essential for understanding mechanisms of infection, replication and pathogenesis. Conventional imaging techniques, although powerful, often lack either the resolution or the multiscale capability required to provide comprehensive structural insight. Synchrotron light sources address part of this limitation by enabling high-resolution imaging across diverse biological samples, with sensitivity at both micro- and nano­scales (Albers *et al.*, 2024 ▸). These capabilities are particularly relevant for studying complex viral structures and their interactions with host systems.

Despite this potential, current synchrotron facilities were not designed or optimized for the stringent requirements of BSL-3 and BSL-4 laboratories. Integrating synchrotron light sources with high-containment environments remains a long-standing challenge because of the need for strict isolation, validated decontamination protocols, and architectural compatibility between containment barriers and beamline instrumentation (Yeh *et al.*, 2021 ▸; Byrum *et al.*, 2016 ▸).

The Orion project addresses this gap by developing a unique integration of synchrotron-based imaging with BSL-3- and BSL-4-compatible environments. Dedicated containment zones will provide the safety standards required for high-risk biological samples while preserving access to the fourth-generation synchrotron radiation generated by SIRIUS. This configuration will allow researchers to investigate the effects of pathogens such as SARS-CoV-2 and mPOX from cellular to small-animal scales within secure biocontainment conditions, without compromising imaging performance.

Here, we present the design concepts and engineering strategies of the SIBIPIRUNA, TIMBÓ and HIBISCO beamlines, focusing on the adaptations required to operate high-performance synchrotron instrumentation inside biocontainment environments. The discussion emphasizes optical and mechanical solutions, redundant containment layers, through-wall loading, and X-ray-transparent barriers that separate biohaza­rdous samples from sensitive beamline components. By combining containment architecture with multiscale imaging, these beamlines aim to establish a framework for safe and effective synchrotron-based biological imaging under BSL-3 and BSL-4 conditions.

## 2. General concept

**Figure 1:**
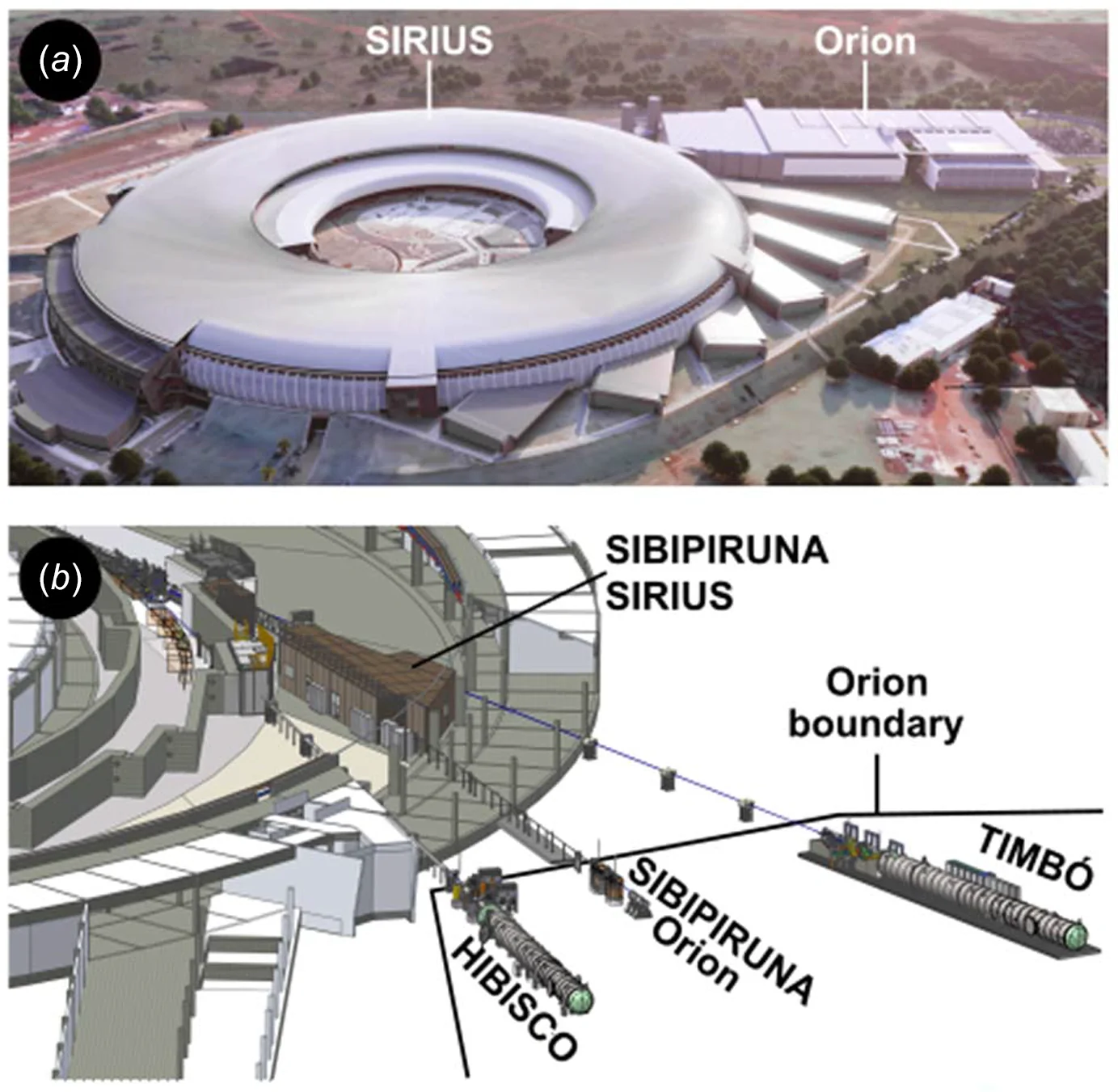
(*a*) 3D rendering highlighting the adjacent SIRIUS and Orion buildings. Credits: Bruno Lucchese/Pedro e Paulo Bruna Arquitetos Associados. (*b*) Schematic representation evidencing SIRIUS and Orion boundaries, the three beamlines, named SIBIPIRUNA, TIMBÓ and HIBISCO, which will have experimental stations built inside Orion, and a complementary experimental station, named SIBIPIRUNA-SIRIUS, which will be built still inside SIRIUS for investigating BSL-2-type cells.

The Orion facility, currently under construction, is located adjacent to the SIRIUS synchrotron light source [Fig. 1 ▸(*a*)]. This proximity was deliberately chosen to enable a unique scientific architecture in which synchrotron radiation can be delivered directly into high-containment biological environments. Rather than physically merging the two buildings, Orion and SIRIUS will remain independent infrastructures, connected only by three dedicated vacuum pipes that transport the X-ray beams from SIRIUS into Orion [Fig. 1 ▸(*b*)]. This solution preserves the strict isolation required for high-containment laboratories while granting access to the brightness, coherence and imaging versatility of a fourth-generation synchrotron source.

Within Orion, three experimental stations will be implemented to cover complementary biological length scales, from single cells to tissues and living organisms. SIBIPIRUNA will be dedicated to cryogenic soft X-ray tomography, enabling nanoscale imaging of individual cells in near-native conditions. TIMBÓ will operate in the tender X-ray regime, extending coherent phase-contrast tomography to cryopreserved tissues and also supporting studies of arthropods under BSL-2 conditions. HIBISCO will use hard X-ray phase-contrast microtomography for organs and small living animals, introducing the organismal scale into the Orion imaging portfolio. Together, these beamlines establish a continuous multiscale framework for investigating infection, host response and disease progression inside a controlled biocontainment infrastructure. Table 1 ▸ summarizes their main technical characteristics, including energy range, sample type, sample size and expected spatial resolution. A complementary station, named SIBIPIRUNA-SIRIUS, will also be built inside the SIRIUS experimental hall, outside the Orion containment domain [Fig. 1 ▸(*b*)]. This station will reproduce the core architecture of SIBIPIRUNA-Orion but will be restricted to non-pathogenic BSL-2 samples. Because it can be implemented ahead of the high-containment stations, SIBIPIRUNA-SIRIUS will serve as both an early scientific platform and an engineering validation environment, allowing optical, mechanical, cryogenic and operational solutions to be tested before deployment inside Orion.

The following sections describe the design rationale of SIBIPIRUNA, TIMBÓ and HIBISCO, emphasizing how each beamline was conceived to match a specific biological scale while complying with the constraints imposed by BSL-3 and BSL-4 operation. Rather than simply extending conventional beamline design into a new building, Orion introduces an integrated strategy in which synchrotron performance, sample workflow and biocontainment are treated as a single design problem.

### 2.1. SIBIPIRUNA beamline

SIBIPIRUNA will be the cellular-imaging pillar of Orion, providing cryogenic soft X-ray tomography (cryo-SXT) for three-dimensional visualization of vitrified cells in near-native conditions (Pereiro *et al.*, 2009 ▸; McDermott *et al.*, 2009 ▸; Schneider *et al.*, 2012 ▸; Harkiolaki *et al.*, 2018 ▸; Lai *et al.*, 2023 ▸). The beamline was conceived to address the growing need for high-resolution imaging of viral infections at the single-cell level (Loconte *et al.*, 2021 ▸; Castro *et al.*, 2023 ▸; Garriga *et al.*, 2021 ▸). By combining soft X-ray absorption contrast with cryogenic preservation, SIBIPIRUNA will enable sub-organelle and viral-particle-scale imaging while minimizing preparation artifacts associated with staining, dehydration or chemical fixation (Mendonça *et al.*, 2021 ▸). This capability is central to the Orion concept, as it provides a direct route to investigate how pathogens reshape cellular architecture under biocontainment conditions.

**Figure 2:**
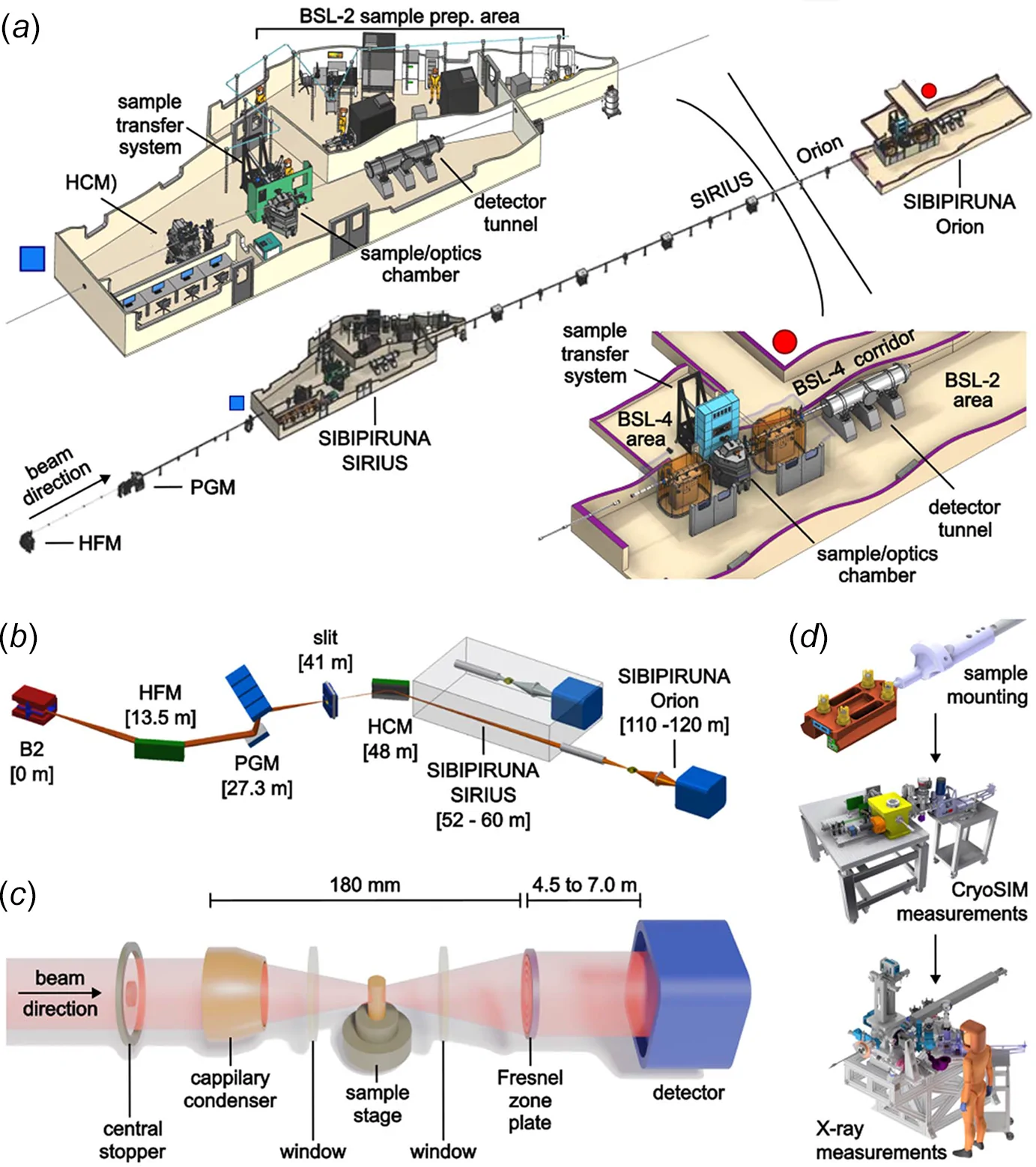
(*a*) Integrated overview of the SIBIPIRUNA beamline, showing photon delivery and the two experimental stations located at SIRIUS and Orion, including their respective sample environments and transfer systems. (*b*) Optical layout based on dipole radiation, plane grating monochromator, and reflective optics for beam conditioning and energy selection in the soft X-ray regime. (*c*) Full-field transmission X-ray microscope configuration, highlighting the condenser optics, sample stage, Fresnel zone plate objective, and detector system used for cryogenic imaging. (*d*) Sample preparation and correlative workflow, illustrating cryogenic preparation, transfer, and integration with complementary imaging modalities.

The integrated design of the beamline is summarized in Fig. 2 ▸. SIBIPIRUNA will include two experimental stations: SIBIPIRUNA-SIRIUS, located inside the SIRIUS experimental hall for non-pathogenic BSL-2 samples, and SIBIPIRUNA-Orion, embedded in the Orion infrastructure for measurements associated with high-containment workflows [Fig. 2 ▸(*a*)]. The SIRIUS station will support early scientific operation, method development, alignment procedures and validation of cryogenic and correlative protocols before their deployment inside Orion. The Orion station will reproduce the core microscope and sample-transfer architecture, but within a hybrid containment strategy in which the high-risk preparation and transfer interfaces are connected to the BSL-4 corridor, while the surrounding beamline instrumentation remains compatible with BSL-2 operation. This arrangement localizes the most restrictive biosafety requirements to the sample pathway, preserving optical stability and operational access.

Optically, SIBIPIRUNA will operate between 300 and 750 eV, an energy range well suited to soft X-ray microscopy of hydrated biological specimens. The layout is based on dipole radiation, a plane grating monochromator and reflective optics for beam conditioning and energy selection [Fig. 2 ▸(*b*)]. This choice provides sufficient flux and field of view for full-field transmission X-ray microscopy while avoiding the thermal and mechanical complexity associated with higher-power insertion-device sources. The optical design also benefits from divergent bending-magnet radiation, which supports the large illumination area required for cellular tomography.

At the microscope level, SIBIPIRUNA follows the full-field transmission X-ray microscopy concept, combining a condenser, cryogenic sample stage, Fresnel zone plate (FZP) objective and detector [Fig. 2 ▸(*c*)] (Pereiro *et al.*, 2009 ▸; McDermott *et al.*, 2012 ▸; Schneider *et al.*, 2012 ▸; Harkiolaki *et al.*, 2018 ▸; Lai *et al.*, 2023 ▸). A parabolic capillary condenser was selected to increase flux efficiency while providing a long working distance, a requirement imposed by the biocontainment-compatible architecture, where the sample environment must be separated from sensitive optics by nested vacuum vessels and thin X-ray-transparent windows. In parallel, the FZP specifications are designed to preserve a target resolution of 45 nm while allowing longer focal distances than typical soft X-ray microscopes, pushing current fabrication limits but increasing clearance for sample rotation and reducing geometric constraints during tomography (Vila-Comamala *et al.*, 2012 ▸). A small-pixel detector will complete the optical chain, matching the microscope magnification required for the target spatial resolution across the operational energy range.

The mechanical design follows the principles of modularization, stability and reduction of exposed components. The condenser, sample and FZP will share a compact, mechanically stable platform, with only the degrees of freedom required for alignment, region-of-interest positioning and tomographic rotation. This minimization is particularly important for operation in a biocontainment context, where components may be exposed to decontamination procedures and where mechanical complexity can increase operational risk. By reducing moving parts and concentrating precision motion close to the optical axis, the station aims to preserve imaging performance while simplifying maintenance and biosafety-compatible operation.

SIBIPIRUNA is not only an imaging instrument but also a complete cryogenic and correlative workflow [Fig. 2 ▸(*d*)]. Samples will be prepared by plunge or jet freezing, transferred under cryogenic and vacuum-compatible conditions, and correlated with a custom-designed structured illumination microscopy (SIM) system developed in-house. This cryo-correlative strategy will support accurate localization of cellular regions of interest prior to soft X-ray tomography and enable multimodal interpretation of infection-related ultrastructural changes. Additional grid- or membrane-based micropatterning strategies will further improve sample positioning and throughput. Together, these elements make SIBIPIRUNA the entry point to Orion’s multiscale imaging framework, linking pathogen biology at the cellular scale to higher-scale tissue- and organism-level information provided by TIMBÓ and HIBISCO.

### 2.2. TIMBÓ beamline

TIMBÓ (Tender X-ray coherent IMaging Beamline for *ex vivo* small animal Organs and tissues) will be the mesoscale-imaging pillar of Orion, bridging the cellular cryo-SXT capabilities of SIBIPIRUNA and the *in vivo* hard X-ray microtomography of HIBISCO. Operating between 3.1 and 20.0 keV with monochromatic illumination, TIMBÓ is designed for cryogenic, high-coherence tomography of tissues, organoids and small organs up to approximately 4 mm, while also supporting in-line imaging of arthropods under BSL-2 conditions. Its scientific mission is to reveal structural correlates of host–pathogen interactions at the tissue scale, including cellular interfaces, stromal organization, vascular remodeling and parenchymal damage, in near-native or controlled biological states (Reichmann *et al.*, 2024 ▸; Shahmoradian *et al.*, 2017 ▸; Sena *et al.*, 2025 ▸).

**Figure 3:**
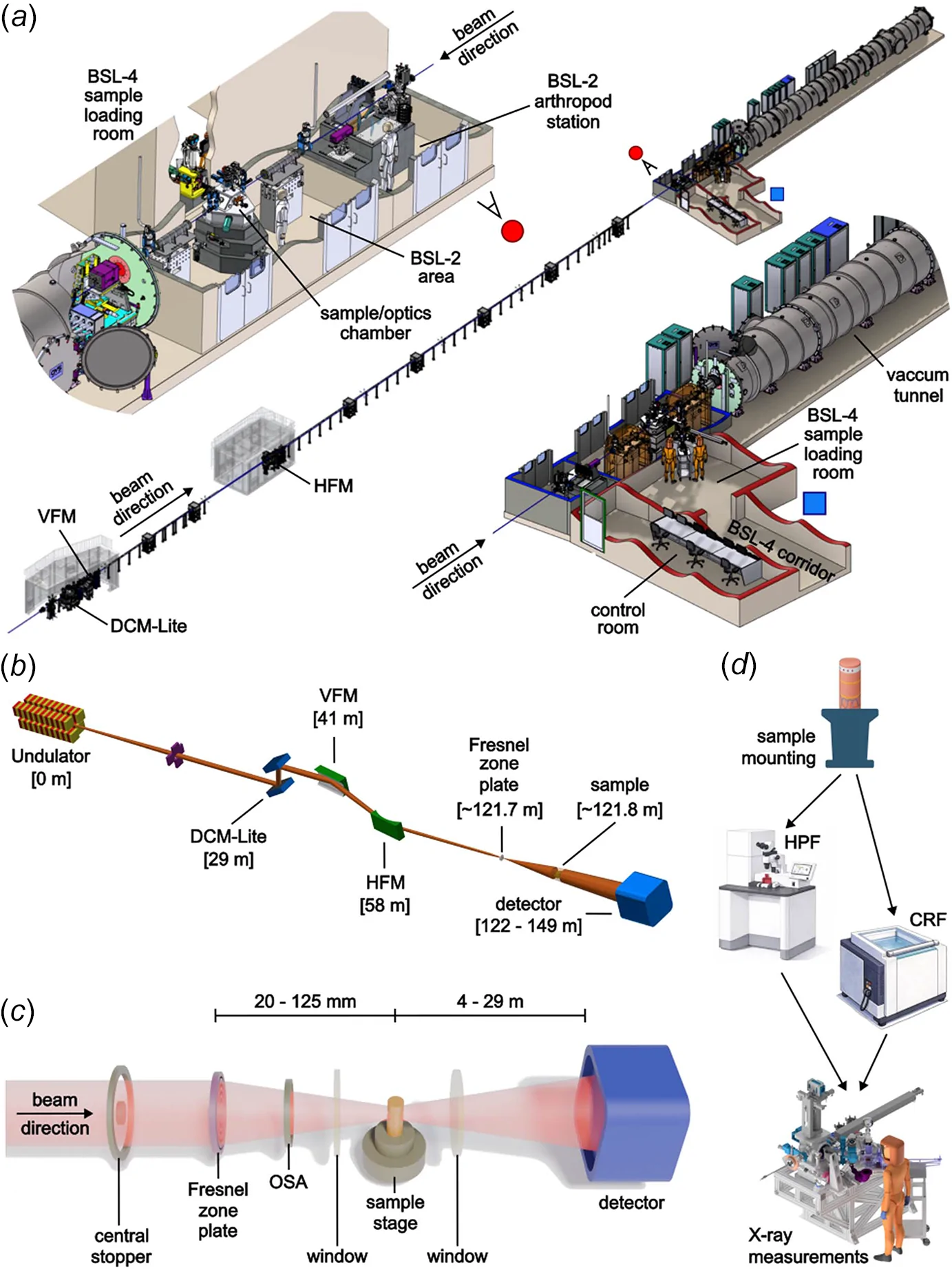
(*a*) Integrated overview of the TIMBÓ beamline, showing photon delivery, beam conditioning optics, and the two experimental branches, including the BSL-2 arthropod station and the high-containment (BSL-4) tomography station. (*b*) Optical layout based on a cryogenic permanent magnet undulator, double-crystal monochromator, and achromatic mirror system for high-coherence beam delivery over extended propagation distances. (*c*) Optical components in the vicinity of the sample position, highlighting the configuration used to shape and deliver the beam for coherence-based imaging modalities. (*d*) Sample preparation strategies for cryogenic imaging, illustrating alternative vitrification methods based on sample size, including high-pressure freezing (HPF) and controlled-rate freezing (CRF).

The integrated architecture of TIMBÓ is summarized in Fig. 3 ▸. In contrast to SIBIPIRUNA, TIMBÓ will be implemented entirely within the Orion facility and will not include a parallel experimental station inside SIRIUS. Nevertheless, its design builds directly on the experience gained at the CATERETÊ beamline (Meneau *et al.*, 2021 ▸), which has served as a testbed for coherent imaging methods and BSL-2 biological measurements at SIRIUS. Downstream, the beamline branches into two experimental configurations: a BSL-2 arthropod station for high-throughput and method-development experiments, and a high-containment tomography station interfaced with the BSL-4 infrastructure. As in SIBIPIRUNA, the design follows a hybrid containment strategy in which the most restrictive biosafety requirements are localized around sample preparation, transfer and measurement, while the surrounding instrumentation remains compatible with stable beamline operation.

TIMBÓ will be fed by a short-period cryogenic permanent magnet undulator installed in a low-beta straight section of SIRIUS, providing high brightness and coherent flux across the tender X-ray range [Fig. 3 ▸(*b*)]. Spectral selection will be performed by the in-house HD-DCM-Lite double-crystal monochromator, whose fixed-exit geometry and high angular stability are essential for long-distance beam propagation to the experimental station (Geraldes *et al.*, 2022 ▸). Downstream of the monochromator, an achromatic mirror system based on side-bounce Rh-coated mirrors (Luiz *et al.*, 2020 ▸) will condition the beam while preserving operation up to approximately 20 keV. This optical architecture is designed to support both large-field propagation-based imaging and coherent nanobeam modalities.

At the sample position, TIMBÓ will support a family of complementary phase-contrast and coherent imaging methods. The optical components positioned near the sample, including beam-shaping elements, order-sorting apertures, sample environment windows and detector geometry, will define how the beam is delivered for coherence-based imaging modalities [Fig. 3 ▸(*c*)]. Parallel-beam X-ray computed tomography (PBXCT) will provide dose-efficient, full-field propagation-based imaging for millimetre-scale specimens and rapid 3D screening (Weitkamp *et al.*, 2010 ▸). Ptychographic X-ray computed tomography (PXCT) will use coherent probes and far-field diffraction patterns to access higher spatial resolution in smaller samples (Dierolf *et al.*, 2010 ▸). Near-field ptychographic X-ray computed tomography (NF-PXCT) will extend ptychographic imaging toward more dose-efficient high-resolution mapping (Stockmar *et al.*, 2015 ▸), while holographic X-ray computed tomography (HXCT) will use spherical-wave illumination and multi-distance phase retrieval to provide sub-micrometric 3D imaging over larger fields of view (Flenner *et al.*, 2020 ▸) (Pacureanu *et al.*, 2025 ▸).

Detector operation will be adapted to each imaging modality and containment constraint. PBXCT may use scintillator-based detection, enabling efficient access to micrometric effective pixel sizes. For PXCT, NF-PXCT and HXCT, large-area detectors and long propagation distances will be used to exploit coherent contrast and flexible sample-to-detector geometries. This multimodal configuration allows TIMBÓ to trade field of view, dose, spatial resolution and acquisition time according to the biological question.

Sample preparation will follow cryogenic strategies adapted to specimen size and morphology [Fig. 3 ▸(*d*)]. Sub-millimetre tissues will be vitrified by high-pressure freezing (HPF), whereas thicker specimens may require controlled-rate freezing (CRF) with cryoprotectants. Samples will be mounted in capillaries or self-standing micrometric pillars and maintained at cryogenic temperature during imaging. A through-wall cryogenic loading system, cryo load-lock, automated transfer interface and temperature-stable sample parking will minimize ice contamination and maximize experimental uptime. These strategies extend the cryogenic workflow established for SIBIPIRUNA to the tissue and organoid scale.

By combining coherent tender X-ray imaging, cryogenic preservation and a containment-aware experimental architecture, TIMBÓ provides the central link in Orion’s multiscale framework. It will connect cellular ultrastructure to organ-level and *in vivo* information, enabling tissue-scale investigation of infection, inflammation and host response under biosafety conditions that are rarely compatible with advanced synchrotron imaging. In this role, TIMBÓ is not only an intermediate-energy beamline but also the platform that transforms Orion from a set of independent instruments into a continuous imaging environment spanning cells, tissues and organisms.

### 2.3. HIBISCO beamline

HIBISCO will be the high-energy, *in vivo* tomography pillar of Orion, completing the multiscale imaging framework established by SIBIPIRUNA and TIMBÓ. While SIBIPIRUNA addresses the cellular scale and TIMBÓ targets cryogenic tissues and organoids, HIBISCO introduces the organismal scale, enabling structural and functional investigation of intact organs and living mammals under BSL-2, BSL-3 and BSL-4 containment. This capability is based on hard X-ray phase-contrast microtomography, long propagation distances, low-dose acquisition strategies and a containment architecture compatible with maximum-biocontainment operation. In this sense, HIBISCO is conceived not only as an imaging beamline but also as a platform for following host–pathogen interactions inside living systems with micrometric sensitivity.

The motivation for HIBISCO arises from limitations in current approaches to high-risk infectious disease research. Clinical CT and magnetic resonance imaging (MRI) lack the spatial resolution and contrast sensitivity required to detect subtle morphological changes in small-animal models, whereas *post mortem* synchrotron imaging, although highly detailed, captures only static endpoints. HIBISCO bridges this gap by enabling micrometre-scale *in vivo* tomography with enhanced soft-tissue contrast and repeated imaging over days or weeks, while respecting strict dose constraints (Bayat *et al.*, 2022 ▸). This opens the possibility of monitoring how infection affects the respiratory tract, how inflammation or vascular leakage evolves spatially, and how organ systems respond as disease progresses. In translational contexts, the ability to extend this strategy to small non-human primates, such as marmosets, further expands its relevance for vaccine development, antiviral testing and immunopathology.

**Figure 4:**
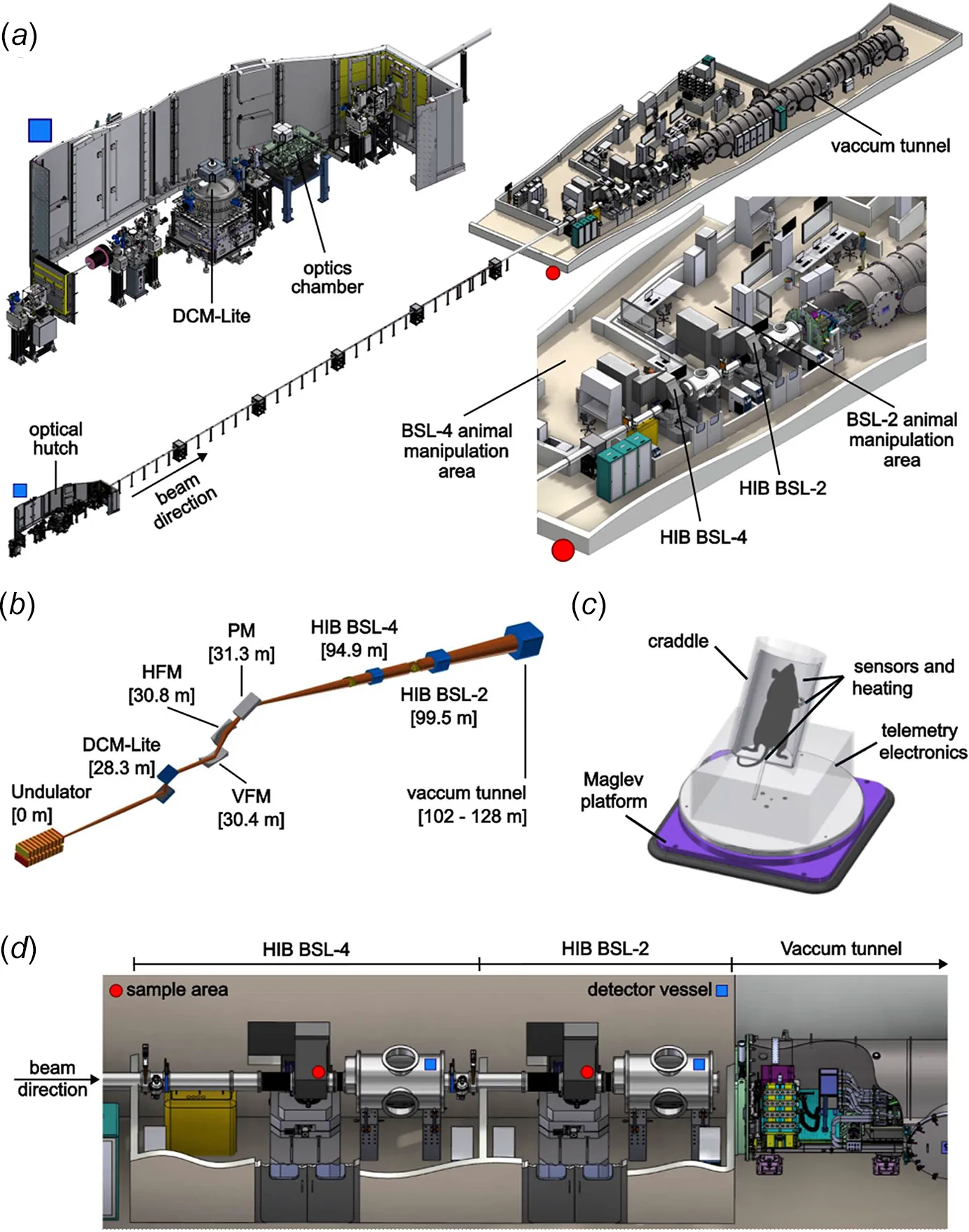
(*a*) Schematic overview of the HIBISCO beamline, showing photon delivery from the source to the *in vivo* experimental stations and the integration with BSL-2 and BSL-4 environments. (*b*) Optical layout, including the monochromator and focusing optics used to condition the high-energy X-ray beam for propagation-based phase-contrast imaging. (*c*) *In vivo* experimental platform, highlighting the animal positioning system, physiological monitoring, and motion-control components required for stable tomography. (*d*) Tomography cabins for BSL-2 and BSL-4 operation, implemented as sealed biocontainment enclosures compatible with animal handling, imaging requirements, and decontamination protocols.

The integrated architecture of HIBISCO is summarized in Fig. 4 ▸. The beamline will operate in the 16–45 keV range, using undulator radiation monochromated by a double-crystal monochromator and conditioned by focusing optics for propagation-based phase-contrast imaging [Fig. 4 ▸(*b*)]. The experimental complex includes two serial tomography stations, one coupled to a BSL-4 animal manipulation area and another to a BSL-2 animal manipulation area. This arrangement allows methodological continuity across biosafety levels while maintaining strict physical separation between containment domains. Downstream of the sample position, an extended vacuum propagation tunnel provides flexible sample-to-detector distances, enabling optimization of field of view, phase contrast, spatial resolution and radiation dose according to the biological application.

The detection and sample-positioning systems are designed around the specific demands of live-animal tomography. sCMOS detectors will be positioned either close to the sample or at the end of the propagation tunnel, depending on the required resolution and contrast regime. The *in vivo* platform combines high mechanical stability with physiological support, integrating precise animal positioning, respiratory and cardiac monitoring, temperature control and anesthetic delivery [Fig. 4 ▸(*c*)]. Mechanical components exposed to the containment environment must withstand repeated decontamination and avoid geometries that retain contaminants. For this reason, the platform emphasizes rigidity, vibration isolation, smooth motion and simplified internal mechanics, preserving tomographic stability while remaining compatible with BSL-4 operational constraints.

A defining constraint of HIBISCO is radiation dose. Conventional synchrotron micro-CT experiments can deliver absorbed doses of several grays, which are incompatible with longitudinal imaging in living animals (McDougald *et al.*, 2017 ▸). HIBISCO is therefore designed around an upper dose target of approximately 100 mGy per tomographic session, supported by radiobiological evidence indicating that such levels can be compatible with repeated imaging when appropriately spaced (Bayat *et al.*, 2022 ▸; Lovric *et al.*, 2013 ▸; Berghen *et al.*, 2019 ▸). Achieving useful image quality at this dose requires synchronizing a fast shutter with respiratory or cardiac signals to illuminate the sample only during selected phases of the physiological cycle (Cercos-Pita *et al.*, 2022 ▸). Combined with efficient scintillators, high-quantum-efficiency detectors and optimized X-ray energies, this strategy concentrates dose on informative projections and reduces motion-related losses.

The biosafety infrastructure is equally central to the HIBISCO concept. The beamline will operate with two independent tomography cabins, one for BSL-2 and one for BSL-4 operation [Fig. 4 ▸(*d*)]. These sealed biocontainment enclosures incorporate controlled ventilation, redundant pressure monitoring, HEPA-filtered air handling and compatibility with chlorine dioxide or vaporized hydrogen peroxide decontamination (Janosko *et al.*, 2016 ▸; Kurth *et al.*, 2022 ▸; Bohannon *et al.*, 2016 ▸). In the BSL-4 configuration, the animal and its immediate environment remain confined within negative-pressure containment, while optical and detector components are protected from routine exposure to aggressive decontamination procedures. This separation is essential to reconcile animal welfare, biosafety compliance and long-term beamline performance.

Through this combination of high-energy phase-contrast imaging, dose-aware acquisition, precision animal handling and maximum-containment infrastructure, HIBISCO will enable biological questions that are inaccessible to existing isolated approaches. It will allow researchers to visualize the progression of respiratory and systemic infections, correlate structural damage with disease evolution, evaluate therapeutic responses *in vivo* and connect organismal phenotypes to tissue- and cellular-scale information obtained with TIMBÓ and SIBIPIRUNA. In this way, HIBISCO completes Orion’s multiscale imaging triad and gives the facility its most distinctive capability, allowing infection to be studied across spatial scales, from cells to tissues and living organisms, within a unified synchrotron-based biocontainment environment.

## 3. Biosafety strategies for BSL-3 and BSL-4 environments

Operating high-containment laboratories (BSL-3 and BSL-4) is a consolidated practice worldwide, yet their integration into synchrotron beamline environments remains largely unexplored (Yeh *et al.*, 2021 ▸; Mora *et al.*, 2016 ▸). Addressing this gap is a central ambition of the Orion project. The facility has been designed through the combined expertise of CNPEM’s Biosafety Office and staff, international biosafety consultants and LNLS engineering teams, integrating containment principles from the earliest stages of beamline conception. This approach builds on the in-house engineering capabilities developed during the construction and operation of SIRIUS, including beamline design, manufacturing and system integration (Liu *et al.*, 2019 ▸; Sanfelici *et al.*, 2019 ▸). It also benefits from advances in critical components such as mirrors, monochromators and experimental stations (Meyer *et al.*, 2022 ▸; Archilha *et al.*, 2022 ▸; Geraldes *et al.*, 2023*a* ▸; Geraldes *et al.*, 2023*b* ▸; Tolentino *et al.*, 2023 ▸).

From an experimental standpoint, two challenges define the biosafety strategy of Orion. The first is the secure transfer of biological specimens from BSL-3 or BSL-4 laboratories to the beamlines while preserving sample integrity and biocontainment (Kong *et al.*, 2022 ▸; Yeh *et al.*, 2021 ▸; Bowman *et al.*, 2024 ▸; Chaki *et al.*, 2024 ▸). The second is the controlled introduction and removal of these specimens from the measurement region without exposing staff, optical components or mechanical systems to biological risk. Meeting these requirements demands sealed interfaces, pressure-controlled transfer pathways, sterilization-compatible materials and redundant containment layers that allow advanced X-ray experiments to proceed while upholding the strict safety standards imposed by high-biosafety operation (Jacobsen & Velisavljevic, 2015 ▸; Kurth *et al.*, 2022 ▸).

### 3.1. Sample workflow to the beamlines

**Figure 5:**
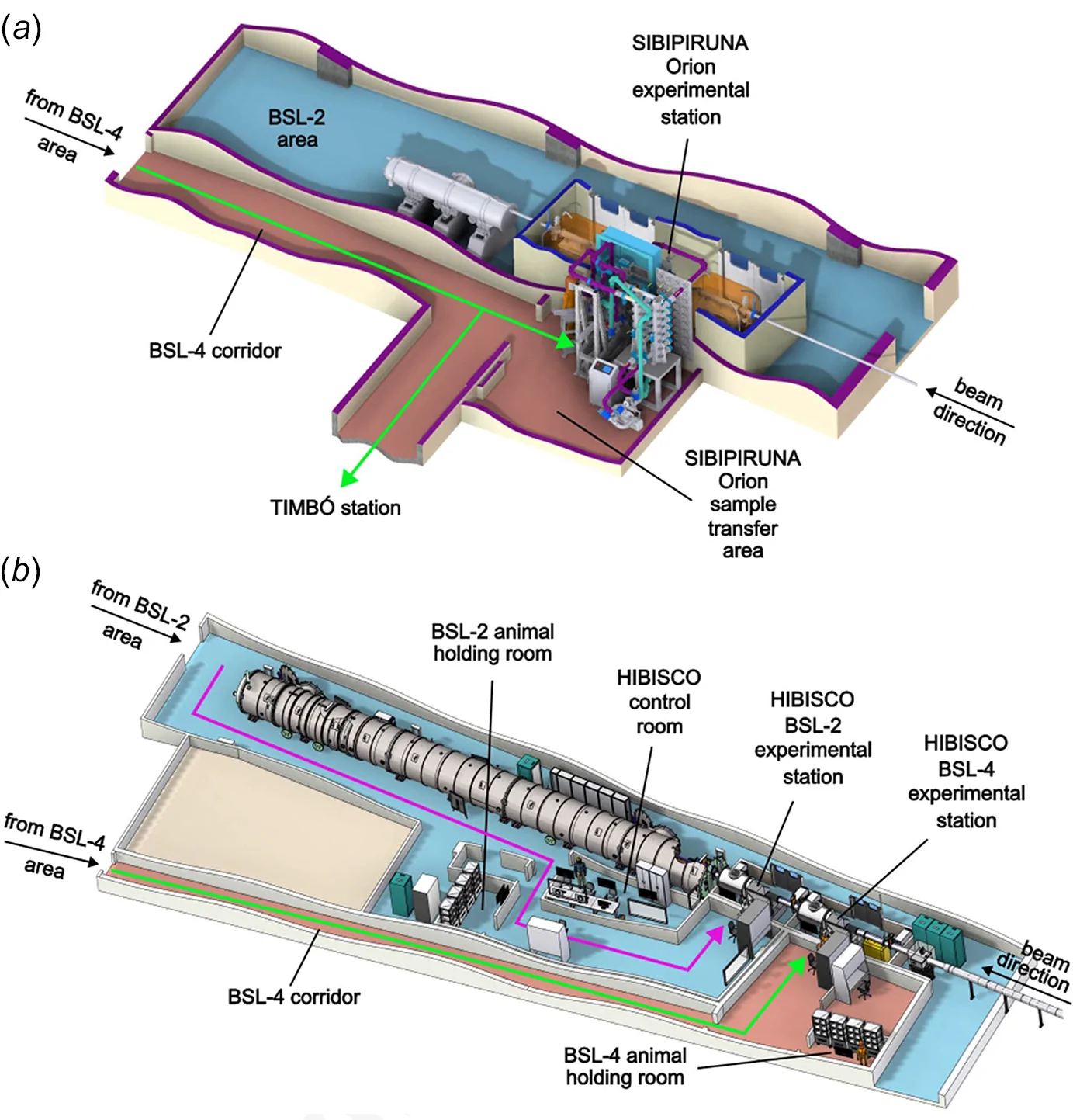
(*a*) Workflow for *ex vivo* samples in the SIBIPIRUNA and TIMBÓ beamlines, illustrating preparation, cryogenic preservation, and transfer through sealed modules toward the beamline sample-loading system under controlled containment conditions. (*b*) Workflow for *in vivo* experiments in the HIBISCO beamline, showing the progression from the vivarium to preparation, anesthesia, physiological monitoring, and transfer into the tomography cabin, with parallel implementations for BSL-2 and BSL-4 environments under strict containment control.

The sample workflow in Orion follows distinct, regulated paths based on the specimen type and the beamline involved (Fig. 5 ▸). For SIBIPIRUNA and TIMBÓ, *in vitro* and *ex vivo* samples are processed in dedicated preparation laboratories before being transferred to the experimental stations [Fig. 5 ▸(*a*)]. Cellular samples are cryopreserved and evaluated using a standalone CryoSIM unit to assess vitrification quality and morphological preservation before synchrotron analysis. Tissue samples follow cryopreservation strategies adapted to their size, geometry and biological composition. Once prepared, specimens are enclosed in sealed cryogenic transfer modules that maintain both low temperature and biocontainment during transport toward the corresponding beamline interface.

HIBISCO follows a separate workflow designed for *in vivo* experiments [Fig. 5 ▸(*b*)]. Animals are transported from the vivarium in individually ventilated containment systems and transferred into preparation areas where anesthesia, physiological monitoring and positioning are established under controlled biosafety conditions. The animal is then introduced into the tomography cabin for imaging and subsequently monitored during recovery. Equivalent but independent workflows are implemented for BSL-2 and BSL-4 operation, allowing methodological continuity across biosafety levels while preserving physical separation between containment domains. In all cases, the workflow is designed to maintain traceability, prevent environmental release and ensure that the specimen reaches the imaging station in a condition compatible with high-quality data acquisition.

### 3.2. Through-wall connection to the beamlines

**Figure 6:**
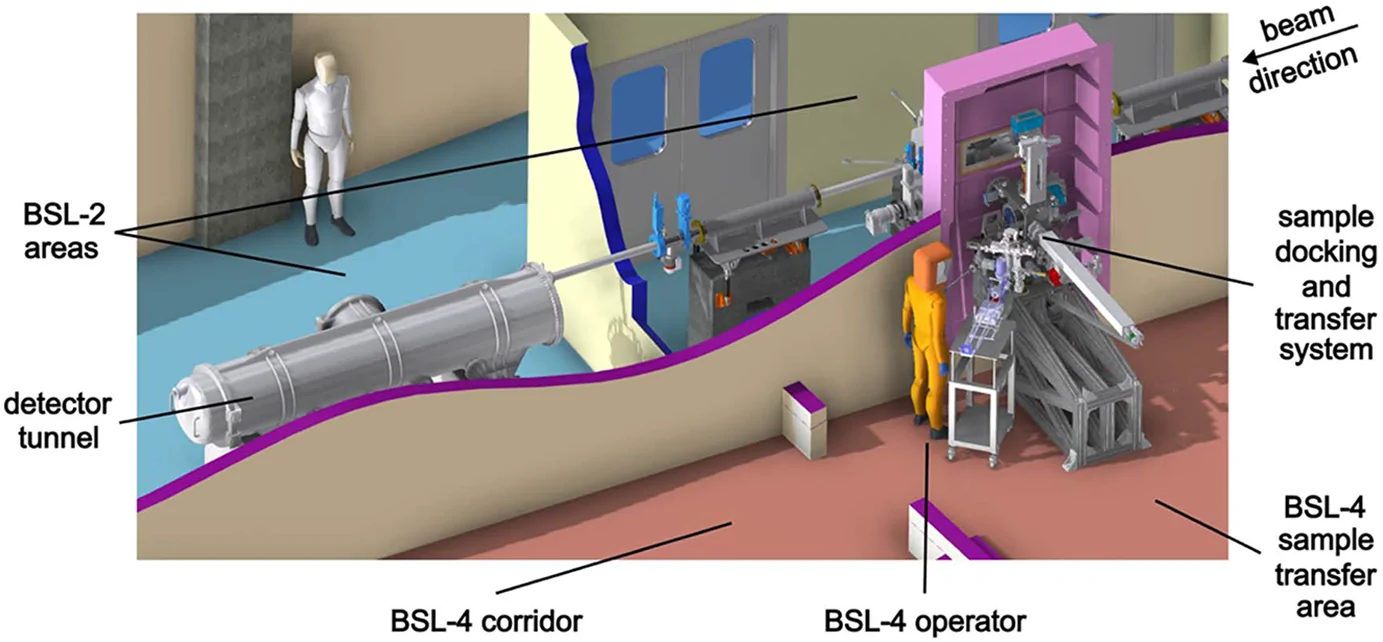
Schematic representation (section view for visibility) of the through-wall architecture implemented in the Orion beamlines for handling high-risk biological samples. The diagram illustrates the physical separation between the BSL-4 environment (orange) and the BSL-2 beamline area (blue), with synchrotron radiation propagating from the beamline into the biocontainment region. Samples are prepared, encapsulated and mounted entirely within the BSL-4 laboratory and introduced into the beamline through a sealed docking and transfer system integrated into the containment wall. This interface ensures that the only element crossing the boundary is the confined sample holder, while all beamline optics, detectors and motion systems remain outside the high-containment area. Downstream of the sample, the transmitted beam is collected in a detector tunnel located in the BSL-2 zone, thereby preventing exposure during decontamination procedures. The architecture minimizes the bioactive volume within the beamline and enables direct coupling between high-containment laboratories and synchrotron instrumentation without compromising biosafety or imaging performance.

A central feature of the Orion biosafety concept is the through-wall connection between high-containment laboratories and synchrotron beamlines (Fig. 6 ▸). In this architecture, infectious or potentially infectious materials are prepared, mounted and conditioned inside the BSL-3 or BSL-4 environment, while the main beamline instrumentation remains outside the high-containment zone. The only element crossing the boundary is the confined sample holder or sealed transfer interface. This strategy preserves the isolation required for high-risk biological work while protecting optics, detectors and precision mechanics from direct exposure to contamination and aggressive decontamination cycles.

For *in vitro* and *ex vivo* measurements at SIBIPIRUNA and TIMBÓ, the through-wall interface enables cryogenic cells, tissues, organoids or engineered biological samples to be introduced into the X-ray measurement region without exposing the surrounding beamline hutch to biohaza­rdous material. The sample chamber acts as the primary containment volume and is separated from sensitive optical components by X-ray-transparent windows and nested vacuum barriers. This configuration reduces the routinely fumigated volume, concentrates the biological risk around the specimen and allows the optical path to remain mechanically stable and clean. These principles are particularly important for nano­scale and mesoscale imaging, where alignment tolerances, wavefront quality and cryogenic stability are critical.

**Figure 7:**
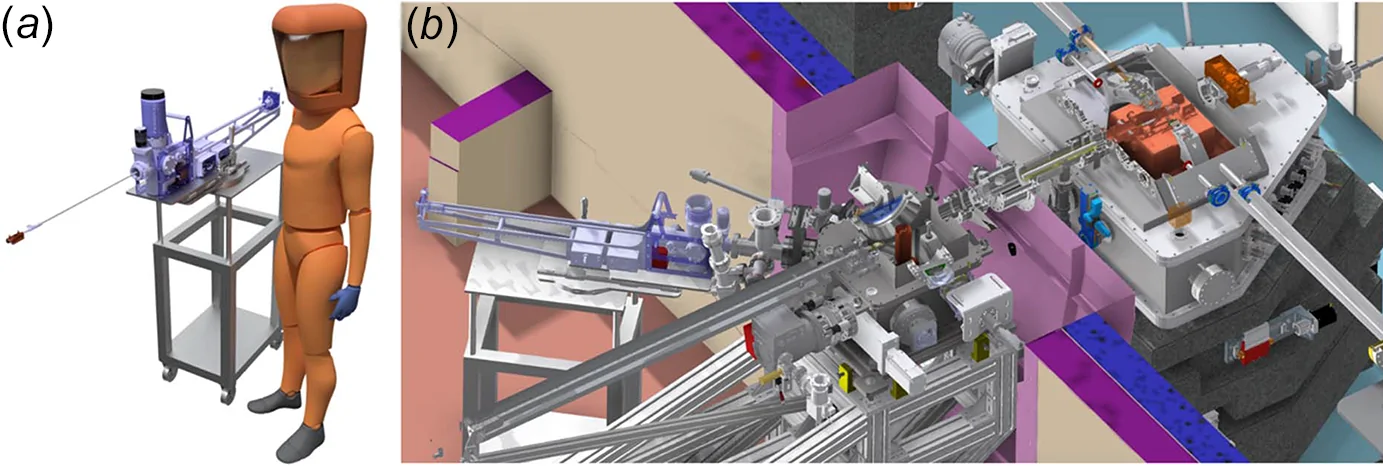
(*a*) Cryogenic transfer shuttle used to transport biological samples under controlled temperature and containment conditions from preparation laboratories to the beamline. (*b*) Docking interface between the cryogenic shuttle and the loading chamber, enabling sealed coupling within a controlled environment. Automated transfer system connecting the loading chamber to the sample environment, enabling insertion and retrieval without compromising containment.

The automated transfer concept is illustrated in Fig. 7 ▸, in which the sample is retrieved from the loading chamber [Fig. 7 ▸(*a*)] and inserted into the sample environment through a protected transfer path that preserves containment [Fig. 7 ▸(*b*)]. This configuration embodies the protective-shell strategy adopted for SIBIPIRUNA and TIMBÓ. The sample chamber and X-ray windows form the primary containment layer, the surrounding optics chamber acts as secondary containment, and the containment buffers and biocontainment enclosure provide a tertiary protective layer. Valves are opened only during loading, transfer or data acquisition, minimizing the affected volume in the event of leaks and reducing the region exposed during fumigation. Standard BSL-4 through-wall sealing concepts are applied at the interfaces between the loading room, enclosure and beam transport lines (Muluneh *et al.*, 2025 ▸; Zhang *et al.*, 2019 ▸).

This layered architecture also supports decontamination and operational recovery. Fumigation lines can be distributed through wall manifolds, while sensitive supports, alignment benches and optical components can be shielded or isolated to reduce unnecessary exposure to chlorine dioxide, hydrogen peroxide or other validated decontamination procedures. Pumping units are connected through HEPA-filtered interfaces and extended piping, and control racks are placed outside the most exposed regions through sealed cable chases. This integrated decontamination strategy, combined with redundant containment barriers, allows SIBIPIRUNA and TIMBÓ to operate safely with biohaza­rdous samples while preserving the performance of high-resolution synchrotron instrumentation (Janosko *et al.*, 2016 ▸; Gao *et al.*, 2025 ▸; Byrum *et al.*, 2016 ▸).

For *in vivo* measurements at HIBISCO, the same containment logic is extended to living animals. The animal remains confined within a sealed tomography cabin that combines primary containment, physiological support and precision positioning. Because live specimens generate aerosols, moisture and metabolic byproducts, the cabin must ensure filtered airflow, thermal stability and condensation control without introducing vibrations that degrade image quality (Bohannon *et al.*, 2016 ▸). The sample stage combines vibration-isolated rotational mechanics with holders compatible with species-specific anatomy and physiological stability. ECG, respiration and temperature are continuously monitored, and anesthesia protocols are integrated into the imaging workflow, with injectable alternatives used when inhaled anesthesia is not feasible (Entenberg *et al.*, 2017 ▸; Jiron *et al.*, 2019 ▸).

Dose management and containment are therefore coupled in HIBISCO. Longitudinal *in vivo* imaging requires limiting absorbed dose while preserving sufficient contrast and spatial resolution. This is achieved by optimizing beam energy, propagation distance, detector efficiency and acquisition strategy, while minimizing non-biological material in the beam path (Donato *et al.*, 2022 ▸; Dierks & Wallentin, 2020 ▸). Components in contact with the specimen must be compatible with BSL-4 decontamination procedures, including vaporized hydrogen peroxide, chlorine dioxide, quaternary ammonium compounds and high-temperature sterilization (McCubbin *et al.*, 2019 ▸). The operational sequence ensures that animals are transferred, prepared, imaged and returned without breaching containment, with positioning, anesthesia control and data acquisition performed remotely whenever required (Shah *et al.*, 2017 ▸).

Across all three beamlines, this standardized through-wall strategy confines biological risk to a small, engineered volume around the specimen, rather than the full experimental hutch. This pocket can be isolated, sterilized or replaced without exposing the broader environment, supported by fast-closing valves, sealed penetrations, pressure differentials, HEPA-filtered interfaces and interlock systems for off-normal conditions. Although samples range from vitrified cells to tissues and living animals, the core biosafety principle remains the same, with the specimen handled under the required containment level while the beamline stays protected, and the interface between domains reduced to a controlled, validated and sterilizable boundary. This reduces the contaminated volume, simplifies decontamination, limits downtime, and preserves personnel safety, regulatory compliance, optical integrity and long-term reliability across SIBIPIRUNA, TIMBÓ and HIBISCO.

## 4. Conclusions

The Orion project establishes a new paradigm for synchrotron-based biological imaging by directly integrating the fourth-generation synchrotron source SIRIUS with BSL-3 and BSL-4 biocontainment environments. By combining advanced photon science with engineered biosafety architectures, Orion addresses a long-standing barrier that has limited the use of high-resolution, multiscale X-ray imaging for studying high-consequence pathogens. The coordinated design of SIBIPIRUNA, TIMBÓ and HIBISCO provides a continuous imaging framework spanning from subcellular ultrastructure to intact organs and living organisms. More than a set of independent beamlines, this triad demonstrates how optical design, mechanical stability, cryogenic workflows, dose-aware imaging and multilayer biocontainment can be co-optimized within a single infrastructure. Key innovations, including through-wall sample transfer, minimized bioactive volumes and redundant containment layers, allow high-performance imaging while preserving biosafety, optical integrity and operational reliability. These capabilities will enable near-native cryogenic tomography of infected cells and tissues, coherent phase-contrast imaging of mesoscale pathology, and longitudinal *in vivo* visualization of disease progression under high-containment conditions. Orion therefore provides a reproducible framework for future facilities seeking to connect advanced synchrotron instrumentation with the study of host–pathogen interactions, tissue remodeling and therapeutic responses under realistic biocontainment constraints.

## Figures and Tables

**Table 1:** General information about SIBIPIRUNA, TIMBÓ and HIBISCO beamlines, such as the technique employed, energy range, type of samples to be measured, sample sizes and expected resolution

Characteristic	SIBIPIRUNA	TIMBÓ	HIBISCO
Technique	Soft X-ray absorption cryo-nanotomography	Tender X-ray coherent phase contrast cryo-nanotomography	Hard X-ray phase-contrast microtomography
X-ray regime	Soft X-rays	Tender X-rays	Hard X-rays
Energy range (keV)	0.30–0.75	3.1–20.0	16–45
Sample type	Cells	Tissues and arthropods	Animal organs, and live rodents and small non-human primates
Sample size	1–10 µm	50 µm–4 mm	4–60 mm
Ultimate expected resolution	45 nm	50 nm (tissue imaging) and 1 µm (arthropods)	10 µm
